# Harnessing Microbial Power for a Sustainable Future Food System

**DOI:** 10.3390/microorganisms13092217

**Published:** 2025-09-22

**Authors:** Andreea Loredana Birgovan (Rhazzali), Elena Simina Lakatos, Lucian Ionel Cioca, Natalia Lorela Paul, Sorin Daniel Vatca, Erzsebeth Kis, Roxana Lavinia Pacurariu

**Affiliations:** 1 Institute for Research in Circular Economy and Environment “Ernest Lupan”, 400561 Cluj-Napoca, Romaniasimina.lakatos@ircem.ro (E.S.L.); natalia.paul@ircem.ro (N.L.P.); sorin.vatca@usamvcluj.ro (S.D.V.); erzsebeth.kis@ircem.ro (E.K.); roxana.pacurariu@ircem.ro (R.L.P.); 2 Academy of Romanian Scientists, 010071 Bucharest, Romania; 3 Department of Industrial Engineering and Management, Faculty of Engineering, Lucian Blaga University of Sibiu, 550024 Sibiu, Romania; 4 Plant Physiology Department, Faculty of Agriculture, University of Agricultural Sciences and Veterinary Medicine, 400372 Cluj-Napoca, Romania

**Keywords:** sustainable food systems, circular bioeconomy, microbial biotechnology, biofertilizers, biofuels, food security

## Abstract

Microorganisms are transforming the way we address sustainability across agriculture, food production, waste remediation, bioenergy, and industrial bioprocessing, offering novel solutions for the food systems of tomorrow. This systematic review examines literature from the last twenty years in order to identify key advances, challenges, and future directions in harnessing microbial systems for sustainable applications, especially those underpinning a resilient future food system. The selected documents allowed a mapping of the most important trends: innovations based on metabolic engineering and omics, the use of integrated biorefineries, and digital monitoring platforms are emerging as catalysts for the transition, while high scaling costs, regulatory challenges, and low public acceptance continue to limit large-scale implementation. The analysis highlights both the major advantages (reducing ecological impact, valorizing waste, diversifying food sources) and the current limits of these technologies, proposing a multi-stakeholder roadmap to accelerate the transition to a circular bioeconomy and a low-carbon and climate-resilient food system.

## 1. Introduction

Global food systems are at a critical inflection point, where demographic pressures, climate change, and natural resource degradation require a profound reform of the way we produce and consume food [1,2,3]. In this context, microorganisms are emerging as a transformative force, capable of redefining processes in agriculture, bioenergy, waste remediation, and human nutrition through sustainable and renewable biotechnologies. The use of traditional fermentation and modern techniques (such as precision fermentation, metabolic engineering, and omics-based fermentation) not only optimizes the yield of microbial valorization products, but also allows rapid adaptation to varied feedstocks, contributing to reducing carbon emissions and food waste [4,5,6].

A deeply integrative approach to microbiotechnology is based on the combined application of genomics, transcriptomics, and metabolomics. This integration provides a solid foundation for the construction of engineered microbial strains with optimal performance, bioprocess efficiency, and operational stability in fermenters. More than simple engineering tools, these strains become optimized biological engines for sustainable transformations that meet the needs [7,8,9].

In parallel, precision fermentation has become a revolution in the contemporary food industry. It allows the recombinant production of alternative proteins, such as those similar to milk or egg proteins, using bacteria, yeasts, or fungi, with a significantly reduced environmental impact. The products obtained can reproduce textures, flavors, and nutritional profiles comparable to conventional ones, contributing to the reduction in animal resource use and carbon emissions. However, the commercialization of these technologies faces important challenges, such as system contamination, the variability of culture media, and the instability of bioreactors, but also consumer reluctance [10,11,12].

Another innovation vector is the development of functional circular biorefineries, which transform agro-industrial residues into valuable products, such as energy, microbial proteins, and biochemicals. Such models are fundamental for both the circular economy and food security, combining bioprocess engineering with advanced digital monitoring technologies and flexible public policies. Only through this type of interdisciplinary synergy, between research, industry, regulators, and society, can a circular food system be built, with low emissions and increased resilience [13,14,15].

In this context, the research approach presented in this paper was built around a central question, more precisely we asked, “what are the barriers and enablers to deploying microbial innovations across five domains (bioremediation, biofertilization, biofuel, biochemical synthesis, and next-generation food systems) in order to address sustainability across agriculture?” This guided the systematic analysis of the literature and the structuring of conclusions regarding the applicability of microorganisms in the transition to a sustainable food system. Therefore, to summarize, in Section 2, we present the methodology, a PRISMA-guided search over two decades, mapping keywords and defining the five areas of activity. In Section 3, the results related to bioremediation, biofertilization, biofuels, biochemical synthesis, and next-generation food systems are presented, along with their mechanisms, benefits, and limitations. In Section 4, discussions on the microbial power for a sustainable food system in the future and the obstacles related to regulation and acceptance of large-scale expansion are presented, and the last section summarizes the conclusions.

**Figure 1:**
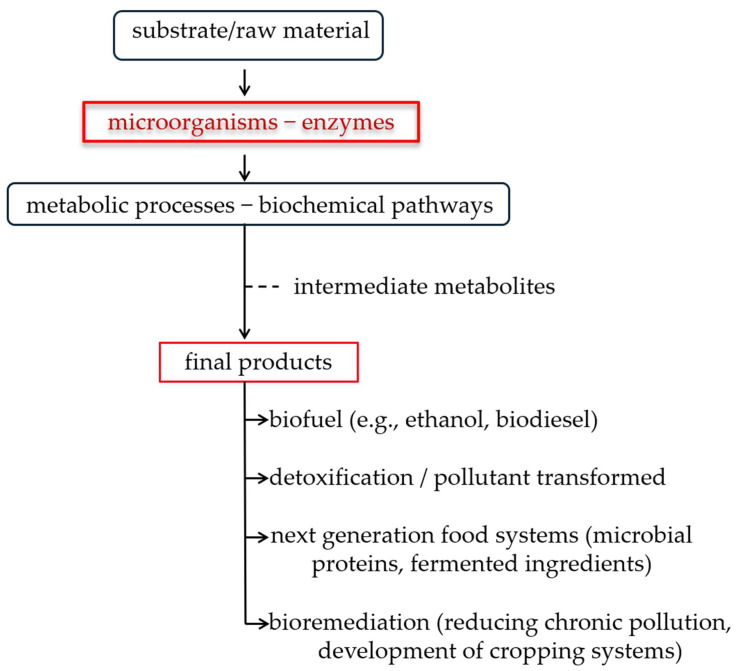
Conceptual diagram of microbial and enzymatic biotransformations.

Figure 1 illustrates how the feedstock or substrate is transformed by microorganisms or enzymes through specific metabolic processes, generating various products of interest. Within this flow, biochemical pathways lead to the formation of intermediate metabolites, which can subsequently be exploited for the production of biofuels (ethanol, biodiesel, hydrogen), for the detoxification of pollutants or environmental bioremediation, as well as for the development of new generation food systems, such as microbial proteins and fermented ingredients. This scheme highlights the central role of microorganisms and enzymes in the transformation of biological resources into sustainable and value-added products, emphasizing the interconnection between bioenergy, environmental protection and food innovation.

Roadmap of the review: this review is structured as follows:

- Section 2 describes the PRISMA-guided methodology and the process of keyword mapping across two decades of studies.
- Section 3 presents the results for each of the five domains, including bioremediation, biofertilization, biofuels, biochemical synthesis, and next-generation food systems, highlighting their mechanisms, benefits, and limitations.
- Section 4 discusses the potential of microbial innovations to drive the transition toward sustainable food systems, addressing regulatory and societal challenges for large-scale implementation.
- Section 5 summarizes the key conclusions and provides insights into future research directions.

## 2. Materials and Methods

To study the complex framework imposed by understanding how microorganisms can change the way we relate to agriculture in terms of sustainability, we conducted a systematic review of the literature. This was performed by querying the Web of Science database, and the preliminary query involved a topic search that integrated the title, abstract, and keywords. The search terms employed included a combination of the following keywords: “microorganisms”, “sustainable food systems”, “circular bioeconomy”, “microbial biotechnology”, “biofertilizers”, “biofuels”, “food security”, “bioremediation”, “biofertilization”, “biofuel production”, “biochemical synthesis”, and “next-generation food systems”. This yielded a total of 30,883 records.

**Figure 2:**
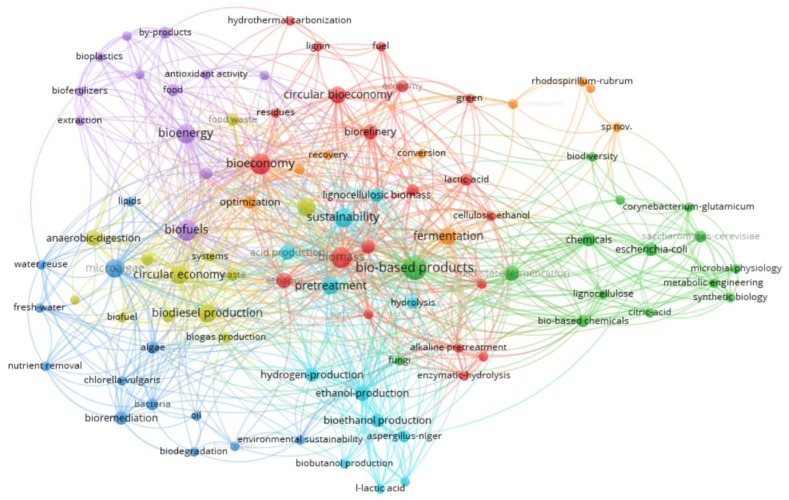
Network visualization map of keywords related to microorganisms and sustainable agriculture.

Later, we filtered the documents based on the following considerations: the publication period (the study is based on the query of documents from the last 20 years, the period of 2005–June 2025), the type of publications (articles and reviews), the language of publication (English), and open access status. The total generated was over 2000 publications, but only 180 met our inclusion requirements. Additionally, we included the key term with the additional AND refinement, “bio-based products”. The additional refinement generated a number of 73 documents that were analyzed in detail for the study of microorganisms in the context of sustainability in the agri-food system transition. We then incorporated these documents for further analysis in VOSviewer software (version 1.6.20) for keyword analysis, selecting the minimum number of keyword co-occurrences as 2. Thus, out of the total number of 486 keywords, 93 met the threshold (Figure 2).

**Figure 3:**
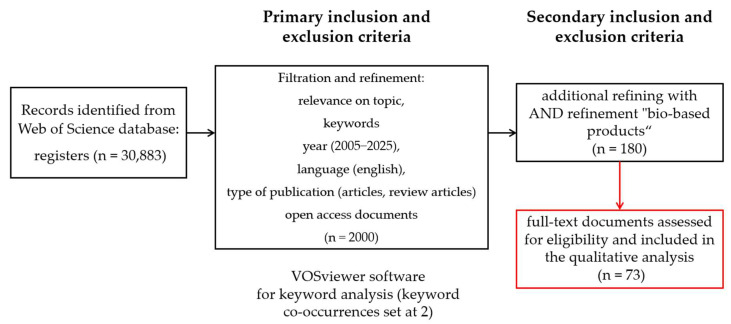
Methodology of research.

Following this analysis, information was further structured into elements corresponding to a context and content analysis. The context elements were structured in accordance with spatial and temporal findings regarding the implementation and role of microorganisms in addressing sustainability in the agri-food sector. Regarding the context elements, they were conceptualized according to five categories, namely bioremediation, biofertilization, biofuel production, biochemical synthesis, and next-generation food systems. Both the review of documents from the specialized literature and their classification into categories were carried out following PRISMA guidelines (Figure 3).

## 3. Results

### 3.1. Context Analysis

Sustainability concepts are frequently addressed in the field of agriculture, but their applicability in close conjunction with aspects related to the use and utility of microorganisms is a debated vision and in transition towards a sustainable agricultural system. The selected documents provide a comprehensive picture of the current state and research conducted, as well as the improvements that can be made in this direction.

**Figure 4:**
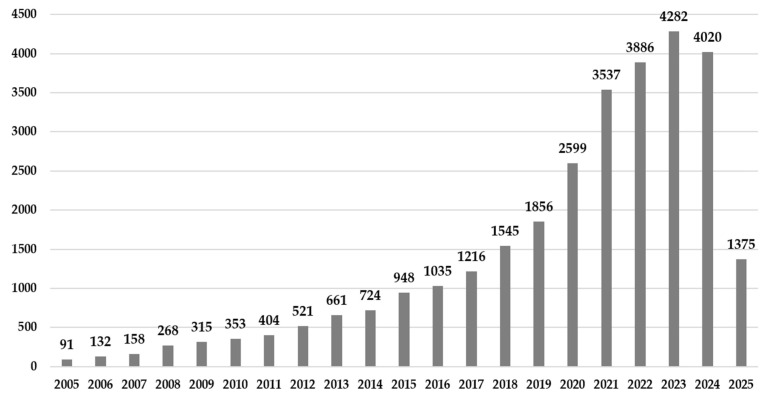
Number of publications related to sustainability in the agricultural sector.

The temporal criteria addressed in the contextual analysis show a concern in analyzing the content of sustainability in the agricultural sector, over the last twenty years (2005–2025). Figure 4 presents the number of publications per year over the last twenty years analyzed in this study, highlighting a notable and relatively recent increase in interest, with a steady upward trend culminating in a peak in 2023.

**Figure 5:**
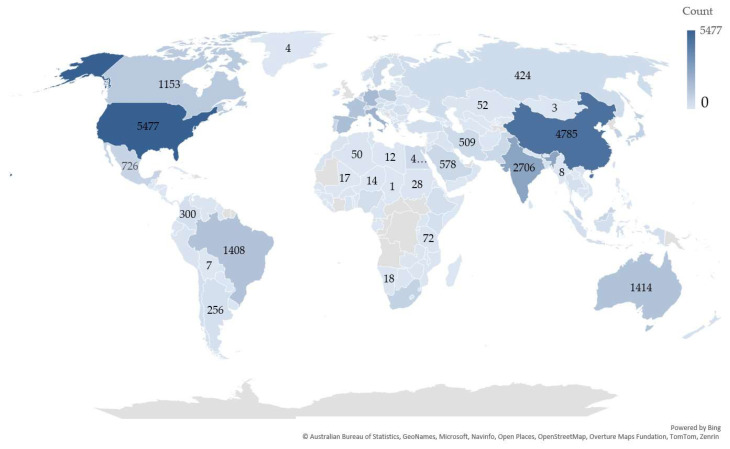
Global distribution of the concerns related to agricultural sector sustainability over the last two decades.

Regarding the contextual analysis from the perspective of spatial terms, we observe in Figure 5, the diversity and popularity of this aspect at a global level. Hotspots are represented by the USA and East and South Asia (China and India, respectively), but the European Union is also among the regions with an increased number of publications in this direction. Among the latter, the top countries with the most publications on this topic include Italy, Spain, Germany, and France. These demonstrate nothing more than the concerns shared at a global level regarding the desire to reform and transition the agricultural system, and implicitly the food system, to a more sustainable, durable, and feasible one in generic terms of the circular economy.

### 3.2. Content Analysis

In the context of the climate crisis and the increasing pressure on natural resources, agriculture is faced with the urgent need to transition to more sustainable practices. One of the most promising research directions in this regard is exploring the potential of microorganisms, especially those in the soil, as essential agents in promoting ecological, efficient, and resilient agriculture. Microorganisms, through their ability to stimulate soil fertility, fix nitrogen, degrade toxic compounds, or induce plant resistance to abiotic and biotic stress, become key elements in a sustainable agricultural system, reducing dependence on chemical inputs and promoting ecological circuits in agroecosystems.

This part of the paper aims to investigate, through a rigorous content analysis, how the specialized literature reflects the contribution of microorganisms to the transformation of agricultural practices from a sustainability perspective. The aim is to identify recurring themes, dominant research directions, and existing gaps in the understanding of the role of microorganisms in agriculture, as revealed by the selected scientific articles. Through this approach, this section contributes not only to a critical mapping of current knowledge, but also to the substantiation of strategic perspectives for the valorization of microorganisms within the framework of future agricultural policies and technologies.

#### 3.2.1. Bioremediation

Bioremediation has become one of the central strategies in efforts to reconcile agricultural production with sustainability imperatives, providing an efficient biological framework for remediating contaminated soils and maintaining the quality of agro-industrial ecosystems. In the last two decades, research in the fields of environmental sciences and agriculture has increasingly highlighted the essential role of microorganisms (bacteria, fungi, and microscopic algae) in the processes of degradation, transformation, and immobilization of chemical and organic pollutants in soils affected by intensive agricultural practices [16,17,18]. As modern agriculture has become increasingly dependent on the use of synthetic fertilizers, pesticides, and herbicides, the accumulation of toxic residues has begun to represent a major threat both to soil biodiversity and to the health of plants, animals, and humans [19]. In this context, microorganism-mediated bioremediation offers an ecological, viable, and scalable alternative to traditional chemical or physical methods of decontamination [16,17,18,20,21].

##### Microorganisms in the Bioremediation Process

Microorganisms implicated in bioremediation exhibit the capacity to metabolize a wide range of toxic compounds, including hydrocarbons derived from petroleum contamination, heavy metals accumulated in soils, and pesticide residues originating from agricultural activities, subsequently converting them into less hazardous or environmentally benign substances. Various species of bacteria (such as *Pseudomonas*, *Bacillus*, *Rhodococcus,* or *Actinobacteria*) have been studied for their ability to degrade polycyclic aromatic hydrocarbons, heavy metals, organophosphorus pesticides, or halogenated compounds, elements frequently found in agricultural soils subject to anthropogenic pressure [22,23,24]. Moreover, mycorrhizae and saprophytic fungi (such as those of the genera *Trichoderma* or *Aspergillus*) have demonstrated an increased potential in immobilizing heavy metals and restoring the microbiological structure of the soil, favoring the natural regeneration of affected ecosystems [25,26,27]. These processes not only reduce environmental toxicity but also contribute to improving soil fertility and restoring beneficial biological activity, creating the premises for a circular and integrative agriculture.

**Figure 6:**
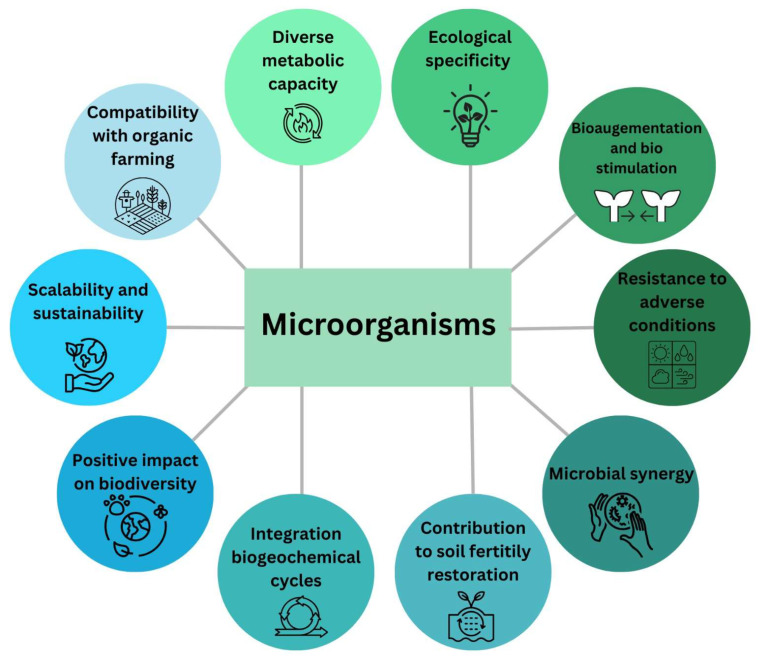
Key aspects of microorganisms in the bioremediation process.

The importance of microbial bioremediation (Figure 6) is also emphasized by current research trends, which increasingly focus on the development of synergistic microbial consortia, adapted to the specific soil and contamination types of various geographical regions [28,29]. Between 2005 and 2025, numerous studies aimed at optimizing environmental conditions to stimulate microbial activity in situ, either through bioaugmentation (the addition of selected microorganisms) or through biostimulation, for instance, improving the nutritional or physico-chemical factors that support native communities of degradative microorganisms. At the same time, advances in the field of metagenomics and molecular biotechnologies have allowed a better understanding of the interactions between microorganisms and pollutants, as well as the identification of genes involved in metabolic biotransformation pathways, paving the way for personalized and efficient applications [30,31,32,33].

Within sustainable agriculture, bioremediation takes on a dual meaning: on the one hand, it contributes to reducing chronic pollution generated by conventional agriculture, and on the other hand, it creates optimal conditions for the development of cropping systems that capitalize on natural resources in a responsible and self-regenerative way. In this sense, bioremediation should not be seen as an isolated practice, but as an integral part of an agroecological vision in which soil health, ecological efficiency, and agricultural productivity are deeply interconnected. Therefore, the use of microorganisms in bioremediation processes not only offers concrete solutions to contamination problems, but also opens innovative perspectives on how the agriculture of the future can be regenerative, adaptive, and environmentally and socially sustainable [21,34,35].

A fundamental aspect of bioremediation in agriculture, closely linked to sustainability, is the role of microorganisms in improving soil fertility [36]. Beyond their ability to reduce or eliminate contaminants, microorganisms directly contribute to the restoration of soil chemical, physical, and biological properties, essential for the long-term productivity of agricultural ecosystems. Nitrogen-fixing bacteria, such as species from the genera *Rhizobium*, *Azotobacter*, or *Azospirillum*, play a central role in the nitrogen cycle, converting atmospheric nitrogen into a form that is assimilable for plants and reducing dependence on synthetic chemical fertilizers [37,38,39]. These biological processes not only provide a constant and environmentally friendly supply of nutrients but also prevent ecosystem imbalances associated with excessive fertilization.

In parallel, soil microorganisms contribute to the mobilization and availability of phosphorus, an essential element for plant growth and development, through the secretion of organic acids and phosphatase enzymes that transform insoluble compounds into accessible forms [40,41,42]. Arbuscular mycorrhizae, part of a complex symbiotic relationship between fungi and plant roots, expand the absorption surface of the root system and improve both the absorption of phosphorus and other nutrients, increasing plant tolerance to abiotic stress conditions, such as drought or salinity [43,44,45]. At the same time, plant growth-promoting bacteria release phytohormones such as auxins, gibberellins, or cytokinins, stimulating root development and, implicitly, the absorption efficiency of soil resources [46,47].

Recent studies have illustrated that the benefits of the association between mycorrhizae and growth-promoting bacteria are not uniform and depend heavily on conditions (soil, phosphorus availability, abiotic stress). For example, in Madagascar, field inoculation with *Rhizophagus irregularis* in rice varieties, in an acidic soil deficient in phosphorus, led to average increases of 28% in yield, 30% in nitrogen, and 39% in phosphorus in grains, but only in the absence of phosphate fertilization; once phosphorus fertilizer was applied, AMF inoculation no longer had a significant effect [48]. Also, a study on 54 maize fields in Switzerland showed that AMF inoculations can produce an average gain of up to 6% in yield, but the variation in response was very large (from −12% to +40%), and the success could be largely explained by soil quality, microbial biomass, or magnesium content [49]. Furthermore, in a water stress experiment, the combination of mycorrhizae and growth-promoting bacteria (AMF and PGPR) increased the uptake of a phosphorus isotope (^33^P) under moderate stress, but under severe stress, the bacteria alone or the combined treatment were more effective than AMF in stimulating phosphorus accumulation in the plant, in part because AMF invested large resources in hyphae/spore formation, which may have energetic–metabolic costs for the plant [50]. From a critical perspective, we can say that these examples demonstrate that the efficiency of AMF and PGPR depends enormously on the level of available phosphorus: when it is very low, AMF have advantages, but when fertilization is applied, the effects diminish. On the other hand, environmental conditions (soil, abiotic stress) and the energy resources of the plant influence whether the benefits can be exploited; for example, investment in fungal structures may reduce growth if the plant already has limited resources. Thus, clear comparisons between treatments (AMF vs. PGPR vs. combinations) are needed under real field conditions, over multiple seasons, as well as attention to cost–energy balance and scalability, so that recommendations regarding use in agriculture are valid and sustainable.

The contribution of microorganisms to soil fertility is also emphasized by their involvement in the formation and stabilization of soil aggregates, by producing extracellular polymeric substances that bind mineral particles and organic matter into a stable structure, favorable to aeration and water retention. This process, essential for preventing erosion and maintaining soil structure, creates an optimal environment for the development of a diverse microbiome, capable of supporting ecosystem functions in the long term. By degrading organic matter and transforming it into humus, microorganisms contribute to increasing the soil’s capacity to store nutrients and water, thus enhancing the resilience of agroecosystems to climate change [48,49,51]. We have summarized, in Table 1, the role of microorganisms in the bioremediation process.

Integrating the role of microorganisms in improving soil fertility into bioremediation strategies offers a complex vision of how biological processes can be harnessed to build a regenerative agricultural system. In recent years, bioremediation has evolved significantly, with an emphasis on the use of microbial consortia for the degradation of recalcitrant pollutants. Recent studies have highlighted the effectiveness of these consortia in removing heavy metals, hydrocarbons, and other persistent pollutants from the environment. For example, combinations of bacterial strains such as *Pseudomonas* sp. and *Bacillus* sp. have demonstrated faster and more efficient degradation of organic contaminants in polluted soils [23,61]. These innovative approaches suggest promising directions for the design of customized bioremediation solutions, tailored to specific pollution conditions. In this approach, bioremediation is not just a cleaning process, but a deep ecological intervention that restores the natural functionality of the soil, promotes microbial biodiversity, and creates the premises for sustainable agricultural production, with minimal impact on the environment. The result is agriculture capable of meeting both production needs and those of conserving natural resources, transforming microorganisms from simple remediation agents into true engines of soil fertility and health [62,63,64].

#### 3.2.2. Biofertilization

Biofertilization is an essential component of sustainable agriculture, a process by which living microorganisms are used to stimulate plant growth and productivity by increasing the availability of essential nutrients in the soil. Over the past two decades, advances in microbiology, biotechnology, and soil science have led to a deeper understanding of the mechanisms by which nitrogen-fixing bacteria, phosphate-solubilizing bacteria, mycorrhizal fungi, and other microbial groups contribute to plant nutrition and soil health. In the context of agricultural intensification and increasing pressure to reduce dependence on chemical fertilizers, biofertilization is emerging as a viable strategy from both an ecological and economic perspective, offering a regenerative alternative that aligns agricultural productivity with environmental protection [65].

##### Microorganisms Involved in the Biofertilization Process

**Figure 7:**
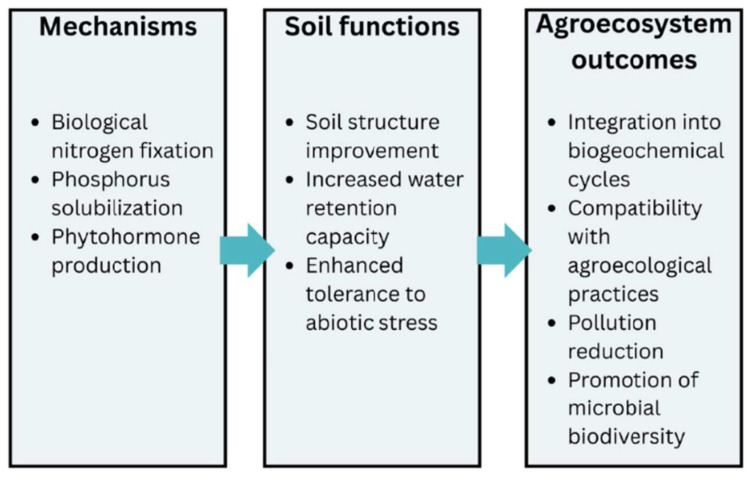
Microorganisms involved in biofertilization.

Microorganisms involved in biofertilization perform multiple functions that converge towards improving soil fertility and the resilience of agroecosystems. Atmospheric nitrogen-fixing bacteria, such as *Rhizobium*, *Azotobacter*, or *Azospirillum*, transform gaseous nitrogen into a form accessible to plants, reducing the need for the application of synthetic fertilizers and preventing the pollution associated with their excessive use. Phosphate-solubilizing bacteria, such as *Pseudomonas* and *Bacillus*, release phosphorus locked in insoluble mineral forms, making it available to plants and thus optimizing the phosphorus cycle. In addition, arbuscular mycorrhizal fungi, through their extensive network of hyphae, enhance nutrient and water absorption, improve soil structure, and increase plant tolerance to abiotic stresses such as drought, salinity, or extreme temperatures [65,66,67,68] (Figure 7).

A major advantage of biofertilization is its ability to integrate microbial processes into natural biogeochemical cycles, creating a dynamic balance between soil, plants, and microbial communities. Recent studies (especially in the last two decades) have highlighted that the use of biofertilizers not only increases crop yields but also contributes to the accumulation of organic matter, increasing water retention capacity and reducing erosion. By stimulating the development of a diverse and active microbiome, biofertilization generates a complex ecological network that improves the resilience of agroecosystems to climate change and anthropogenic disturbances [42,64,65,69].

Recent advances in genomics and molecular biotechnology have opened new perspectives for the personalization of biofertilizers depending on the specific pedoclimatic conditions and crop type. Research published in recent years has demonstrated that microbial strains selected on the basis of metabolic efficiency, stress resistance, and ecological compatibility can be combined in optimized consortia for maximum results. Thus, biofertilization becomes not only an applied technology, but also a field of scientific innovation, located at the intersection of agronomy, ecology, and biotechnology [70,71,72,73]. We have summarized, in Table 2, the role of microorganisms in the biofertilization process.

Overall, biofertilization is emerging as a strategic solution for transforming agricultural practices towards sustainability, offering multiple benefits that go beyond simply increasing production. By reducing chemical inputs, maintaining soil health, and promoting ecological resource management, this approach contributes to shaping a resilient agricultural system capable of responding to environmental challenges and the food demands of a growing global population. In this context, the role of microorganisms as biofertilizer agents is no longer an auxiliary one, but central, defining how the agriculture of the future will manage to combine productivity with ecological responsibility [80].

Recent innovations in the field of biofertilizers include the development of multi-strain formulations that combine plant growth-promoting bacteria with arbuscular mycorrhizal fungi [81]. These combinations can significantly improve nutrient uptake and stress resistance of plants, respectively, and constitute innovative approaches, suggesting promising directions for the design of customized solutions.

#### 3.2.3. Biofuel Production

**Figure 8:**
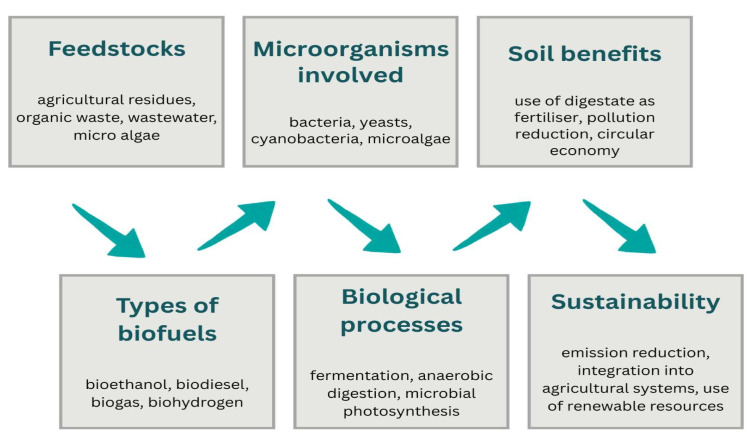
Role of microorganisms in sustainable biofuel production.

Biofuel production is one of the most dynamic research and application directions in the context of the global transition to renewable energy sources, and microorganisms play a central role in this process. In the last two decades, interest in bioenergy has increased with the background of the rising costs of fossil fuels, the pressure to reduce greenhouse gas emissions, and the need to develop energy systems integrated with sustainable agriculture. The use of microorganisms in biofuel production is based on their ability to transform renewable biological resources, including agricultural waste and organic residues, into liquid, gaseous, or solid fuels with high energy value, while reducing pollution and contributing to the circular economy [82,83,84,85,86,87] (Figure 8).

##### Microorganisms in the Biofuel Production Process

Significant progress has been made in the use of bacteria, yeasts, and algae to produce various types of biofuels. Bacteria such as *Clostridium acetobutylicum* are used for the fermentation of acetone, butanol, and ethanol, while yeasts such as *Saccharomyces cerevisiae* are being intensively studied for the production of ethanol by fermenting sugars from lignocellulosic biomass. This biomass, derived from plant residues, straw, husks, or stems, is an abundant resource that does not compete with food production, making it ideal for obtaining second-generation bioethanol [80,81,82,88]. For example, the efficiency of microbial consortia adapted for the simultaneous hydrolysis and fermentation of lignocellulose has achieved superior yields compared to conventional processes [83,84,85].

In parallel, research on microalgae, such as *Chlorella vulgaris*, *Nannochloropsis*, and *Scenedesmus obliquus*, has shown remarkable potential for biodiesel production, due to their high lipid content, which can exceed 50% of the dry weight of the biomass. These microalgae can be cultivated on wastewater or in photobioreactor systems, using carbon dioxide from industrial sources, which transforms the biofuel production process into an efficient method of carbon capture and valorization [89,90,91,92].

In addition to bioethanol and biodiesel, biogas production by anaerobic digestion mediated by microorganisms is a mature technology, but with significant potential for optimization. Anaerobic microbial consortia, including hydrolytic, acidogenic, acetogenic bacteria, and methanogenic archaea, convert organic matter from animal manure, crop residues, and other agricultural by-products into methane, which can be used as an energy source for heating, electricity generation, or as biomethane for injection into natural gas networks [93,94,95,96]. In a recent study, the use of bioaugmentation with selected methanogenic strains was demonstrated to lead to an increase in methane production from cattle manure, demonstrating the importance of optimizing microbial communities in such systems [89,90,97].

Another emerging area is advanced biofuels, such as biohydrogen, obtained by dark fermentation or photofermentation [98]. Microorganisms such as *Enterobacter aerogenes*, *Clostridium butyricum,* and purple photosynthetic bacteria are capable of producing hydrogen by converting carbohydrates from agricultural waste. In addition, recent research is exploring the use of genetic engineering to create strains with high yields and increased resistance to variable conditions, essential for industrial-scale application [99,100,101]. We have summarized, in Table 3, the role of microorganisms in the biofuel production process.

Biofuel production through microbial processes has a dual advantage in the context of sustainable agriculture: it capitalizes on waste streams that would otherwise contribute to pollution and provides a source of renewable energy, reducing dependence on fossil fuels. The integration of these technologies into farms and agricultural cooperatives can create low-emission production systems, where residues become resources and the energy generated is reinvested in the agricultural production chain. Diversification and refinement of microbial technologies for biofuel production supports the idea that these processes can become the backbone of sustainable and circular agricultural systems. Advances in bioprocesses, optimization of culture conditions, development of synergistic microbial consortia, and application of advanced biotechnologies are converging towards a vision in which agriculture is not only an energy consumer, but also an active producer of renewable energy, with long-term economic, ecological, and social benefits [107,108].

In terms of innovations, the production of biofuels from lignocellulosic biomass has been improved by using oleaginous microorganisms that can accumulate lipids similar to those in vegetable oils under stress conditions. Recent studies have highlighted the potential of these lignocellulosic substrates for biodiesel production. For example, the use of agricultural waste for biodiesel production has been investigated, showing that oleaginous microorganisms can accumulate lipids under controlled conditions [113,114]. These innovations highlight the potential of microbial biofuels as a sustainable and scalable source of energy, opening new directions for future research in bioenergy.

#### 3.2.4. Biochemical Synthesis

**Figure 9:**
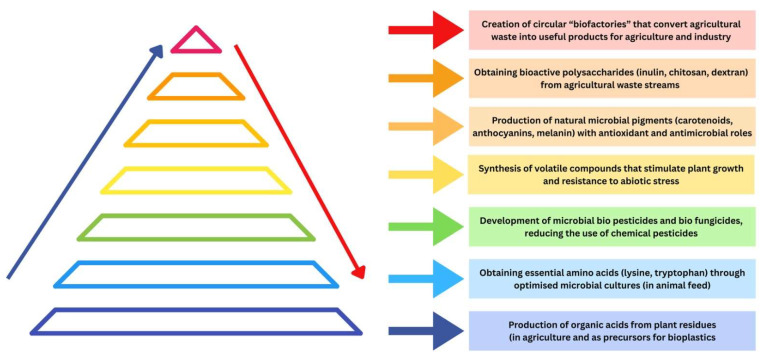
Importance of microbial biochemical synthesis in sustainable agriculture.

Biochemical synthesis by microorganisms has become an increasingly relevant component of strategies for sustainable agriculture. At the confluence of biotechnology, circular economy, and agricultural ecology, the process of producing value-added compounds (such as organic acids, biodegradable polymers, nutraceuticals, biopesticides, and bioingredients) has considerable potential to transform agricultural resource flows into a regenerative and profitable system [115,116,117] (Figure 9).

An example in this regard is the microbial production of organic acids such as citric, itaconic, lactic, and acetic acid [118,119]. These molecules, intended for the food, pharmaceutical, textile, and organic agriculture industries, are generated through controlled fermentation with fungi *Aspergillus* spp., *Lactobacillus*, or *Acetobacter* bacteria, transforming unvalued raw materials such as fruit waste or vegetable residues into products with high economic value and low environmental impact [113,120].

Microorganisms are also increasingly used to produce compounds with nutritional or medical value (antibiotics, anticancer, antiviral, antiparasitic, or antimicrobial), through metabolic engineering and mixed cultures [121]. Examples include substances such as ivermectin, resveratrol, clavulanic acid, or curcuminoids, obtained in yeast (such as *S. cerevisiae*) or bacteria (such as *E. coli* or *Bacillus subtilis*), often using agro-industrial waste as a source of raw materials, which supports the circular economy [121,122,123].

Also, agro-industrial waste can be transformed into microbial proteins used in animal feed, within sustainable “waste-to-protein” systems, which solve both the problem of waste management and that of food security [124]. At the same time, the production of fermentative biopolymers, such as dietary fibers, exopolysaccharide gum, betaglucan, pullulan, xanthan, curdlan, or bacterial cellulose, made by microorganisms such as *Aspergillus*, *Bacillus*, *Xanthomonas,* or *Aureobasidium*, is gaining popularity. These products, used as stabilizers, water retention agents, or texturants in various industries, can be obtained on alternative agricultural substrates, reducing costs and environmental impact [118,119,125,126].

In the last two decades, the development of biochemical synthesis technologies has experienced a remarkable acceleration due to advances in applied microbiology, biotechnology, and synthetic biology. Microorganisms are exploited not only for the production of traditional compounds, but also for complex molecules, with a role in agriculture, nutrition, health, and advanced materials [114,127,128]. A relevant example is the production of organic acids with agricultural value, such as lactic acid and succinic acid, obtained by microbial fermentation of plant residues [129]. These compounds serve both as biopreservatives and as precursors for bioplastics, thus contributing to reducing dependence on petroleum resources. Similarly, the production of essential amino acids (lysine or tryptophan) by optimized cultures of *Corynebacterium glutamicum* or *Escherichia coli* has allowed the production of nutritional supplements for animal feed, with a positive impact on agricultural yield [130,131,132].

##### Microorganisms in Biochemical Synthesis for Sustainable Agriculture

In addition to these examples, biochemical synthesis has begun to be used for the development of microbial biopesticides and biofungicides. Bacteria such as *Bacillus thuringiensis*, *Pseudomonas fluorescens*, and *Streptomyces* spp. can produce secondary metabolites active against agricultural pests and pathogens, reducing the need for chemical pesticides. In parallel, metabolically engineered yeasts and bacteria can produce beneficial volatile compounds that stimulate plant growth and resistance to abiotic stress. These innovations align the goals of sustainable agriculture with the requirements of food safety and environmental protection [133,134,135].

Another emerging area is the use of microorganisms for the synthesis of natural pigments with antioxidant and antimicrobial roles, such as carotenoids, anthocyanins, or microbial melanin, which can be used both in the food industry and in plant protection. Also, the synthesis of bioactive polysaccharides (including inulin, chitosan, and dextran) by bacterial and fungal cultures has been adapted to valorize agricultural waste streams, reducing production costs and ecological footprint [129,130].

Recent research has shown promising progress in transforming agro-industrial residues, wastewater, and food waste into valuable microbial biochemicals and bioplastics, but these advances reveal several critical trade-offs. For example, a 2025 analysis of the production of poly(hydroxyalkanoate) biopolymers (PHAs) from agricultural residues showed that while the use of residual biomass can significantly reduce feedstock costs, the production cost of PHA remains higher than that of conventional plastics, and the isolation and purification of the product is energy-intensive [136]. Another study highlighted the potential of wastewater as a sustainable substrate, but showed that PHA yields depend largely on wastewater composition, organic load, and fermentation conditions, with large variations between batches [137]. Integrating these processes into modern agricultural systems allows for the creation of circular “biofactories” where raw materials come from plant residues or agro-industrial by-products, and the resulting products return to the agricultural chain in the form of fertilizers, soil improvers, biological protection agents, or biodegradable materials. This approach transforms microbial biochemical synthesis into a pillar of the bioeconomy, capable of supporting the transition to low-emission, competitive and climate-resilient agriculture [138]. Thus, microbial biochemical synthesis is not limited to obtaining nutrients but plays a key role in modernizing sustainable agriculture. From organic acids and biopolymers to bioplastics and functional ingredients, these processes create a regenerative production chain, in which microorganisms are engines of ecological and economic transition. We have summarized, in Table 4, the main role of microorganisms in biochemical synthesis for sustainable agriculture.

The synthesis of biopolymers, such as polyhydroxyalkanoates (PHAs), from food waste has been explored as a sustainable method for producing biodegradable plastics. These advances highlight the role of microorganisms as drivers of the circular bioeconomy, providing clear directions for future research in sustainable bioproduction.

#### 3.2.5. Next-Generation Food Systems

The discussion of next-generation food systems has evolved, especially in the last two decades, beyond simple technological concepts, becoming an interdisciplinary field in which biotechnology, agroecology, and public policy converge to provide viable alternatives to conventional animal production chains. Two technical branches have emerged as pillars of this transformation: precision fermentation, the use of engineered microbial hosts to produce proteins and ingredients identical to or similar to those of animals, and single-cell protein (SCP) production, dried microbial biomass used as a concentrated source of protein for human and animal food. These approaches not only offer potentially more efficient ways to provide protein, but also allow the valorization of agricultural residues and secondary flows, thus contributing to the circularity of resources in agriculture [138,139,141,144].

##### Key Innovations for Next Generation Food Systems

Technologically, key innovations can be grouped into three broad directions that determine the feasibility and impact of these systems. The first direction is microbial host engineering: progress in metabolic engineering, synthetic biology, and omics has allowed the redesign of yeasts, bacteria, and even some microalgae to produce complex proteins, enzymes, lipids, or other compounds with food value at increased yields and purities. Explicit examples include industrial platforms using *S. cerevisiae* or *Pichia pastoris* for functional proteins and dairy equivalents, as well as bacterial strains optimized for the rapid production of SCPs on unconventional substrates [140,141,145,146].

The second direction is represented by bioprocessing: the development of specific bioreactors, fed-batch and continuous fermentation strategies, downstream technologies for extraction and purification, and combined systems (consolidated bioprocessing) that combine hydrolysis and fermentation in compact cycles [147,148,149,150,151].

The third direction is feedstock integration: the shift from expensive food substrates to non-food competing sources (agricultural residues, agro-industrial flows, captured CO_2_, or treated wastewater), which links technology directly to the goals of agricultural sustainability and circular economy [10,152,153].

##### Challenges in Implementing Next Generation Food Systems

The transition from the laboratory to commercial scale remains the main challenge. Scaling up means more than increasing the reaction volume; it involves fine control of product quality, genetic stability of strains, significant capital costs for scale-up fermentation facilities, high-volume substrate logistics, and efficiency in downstream processes that often dominate the final cost. Many players in this sector scene signal the scale-up difficulties: namely, the lack of compliant production capacity, specific facility requirements, and financial barriers mean that many promising innovations remain at the pilot or demonstration level. Optimizing large facilities can significantly reduce costs, but to achieve parity with conventional sources, both yield improvements and massive infrastructure investments are needed [154,155,156,157].

Another key set of barriers is regulatory and social. In terms of regulation, markets vary greatly; some jurisdictions have opened fast-track pathways for novel foods and have authorized products derived from precision fermentation or cultured products, while others, particularly some European regions, maintain long and complex procedures that slow down market access. In parallel, consumer perception and social acceptability play a critical role. Communication of safety, ethics, and environmental benefits influences adoption, and transparency regarding microbial processes and the provenance of raw materials is a determining factor. Therefore, the commercial success of next-generation systems depends not only on technology, but also on regulatory clarity and effective communication and labeling strategies [158,159,160,161].

##### Recommendations for Overcoming the Challenges Associated with Next Generation Food Systems

In the face of these challenges, packages of interventions that can act as enablers for the sector are recommended. The first element is public funding directed towards production infrastructure (pilot biorefineries, shared-use facilities) and applied research for scale-up [162]. In this regard, public investments can reduce risk for private investors and accelerate the maturation of technologies. The second element is the harmonization of regulations and clarification of authorization pathways for novel foods and ingredients obtained through biotechnology, together with safety and labeling standards that increase consumer confidence [163]. The third element refers to supporting regional supply chains for non-competitive substrates, through agricultural policies that encourage the valorization of residues and the creation of synergies between farmers and fermentation plants [155,156].

Examples of good practice include government initiatives and framework programs that allocate grants or tax incentives for fermentation facilities, as well as national strategies for alternative proteins that include funding for research and infrastructure. For instance, in the specialty ingredients category, precision fermentation has already enabled the commercial production of dairy proteins without the use of animals, demonstrating technical feasibility and market acceptance for premium segments [164,165]. In the food and feed biomass category, SCPs produced from bacteria, yeasts, or microalgae have been widely tested as animal feed ingredients or protein supplements, with life cycle assessment (LCA) studies indicating the potential to reduce the carbon footprint when the process uses non-competitive substrates. There are also numerous pilot initiatives that use local agricultural waste streams to feed regional-scale fermentations, thus closing the nutrient cycle at the farm or community level [141,158,166].

A key chapter is the assessment of sustainability throughout the life cycle. LCAs for products made by precision fermentation or SCP highlight heterogeneous results depending on two key variables, the source of the substrate and the efficiency of the bioprocess (yield, energy for downstream). When the substrate is a local waste stream and the processes are optimized, the potential for reducing emissions and land use is substantial. On the other hand, if the feedstock is derived from competing food materials or if the downstream is very energy intensive, the benefits are considerably reduced. This explains why some policy planning recommends incentives for the use of second-generation substrates and investments in downstream research to minimize energy consumption [158,159,167].

Looking ahead, the prospects for next-generation food systems are shaped by some converging forces: reducing technology costs (through strain and process optimization), increasing production capacity (through infrastructure and public/private investment), and maturing regulatory frameworks and social acceptance. If these conditions are met, precision fermentation and microbial protein-based technologies can become complementary and integrated components of more resilient agricultural systems, providing farmers with new markets for raw materials and contributing to decarbonization goals. At the same time, realizing this potential will require deliberate policies that unite agriculture, bio-manufacturing, and food security in a coherent framework [168,169,170].

**Figure 10:**
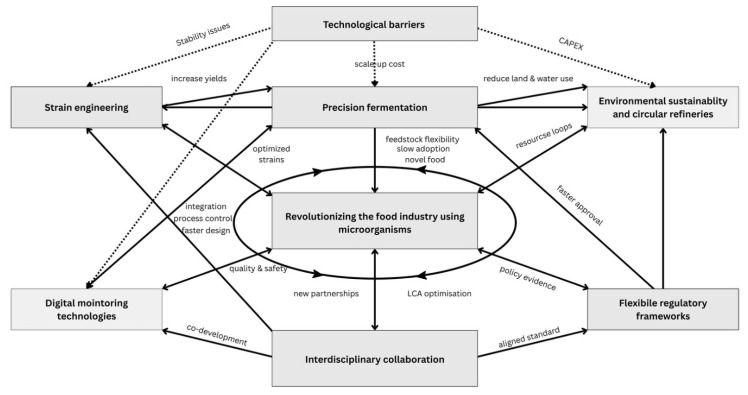
Main barriers in the implementation of next-generation food systems.

In other words, next-generation food systems based on precision fermentation and microbial protein represent a significant opportunity to transform agriculture towards more sustainable and circular models as can be seen in our proposal in Figure 10. The technology exists and is advancing rapidly; the major obstacles currently relate to scaling, economics, and regulation, and addressing them requires the right combination of technological innovation, infrastructure investment, and clear public policies to encourage adoption and protect consumer safety and trust.

The use of microbial proteins and precision fermentation to develop alternative and functional foods is an emerging trend in sustainable food systems. Precision fermentation can produce milk and egg proteins, providing alternatives to animal products. These innovations open up prospects for more sustainable and resilient food systems, stimulating future research into alternative and nutritious foods.

We have summarized, in Table 5, the main advantages and disadvantages of microorganisms in the processes preceding the transition to a sustainable agricultural system.

For guidance, Table 6 summarizes the current status and maturity of microbial products in different categories. It compares products already commercialized with those in pilot or development stages, providing a clear picture of the technological readiness of the field. The categories include biofuels, microbial proteins, pigments, bioplastics (PHA), biofertilizers, and food ingredients. This overview highlights both established applications and emerging innovations, demonstrating the growing potential of microbial biotechnology in the industrial, environmental, and food sectors.

## 4. Discussion

The integration of microorganisms into agricultural and food sustainability strategies is a central direction in the transition towards production systems with reduced environmental impact [5,178]. The last two decades have witnessed a remarkable diversification of microbial applications, from their traditional role in bioremediation and biofertilization processes, to innovation in emerging areas such as the production of advanced biofuels, the biochemical synthesis of high-value compounds, and the development of next-generation food systems based on precision fermentation and microbial proteins [30,31,108,146,163,179]. This application diversity highlights the metabolic flexibility of microorganisms and their ability to transform diverse resources, including agro-industrial wastes, into sustainable products with high economic and ecological value [57,178,180,181].

The integrated analysis of the role of microorganisms in agricultural sustainability reveals a convergence between technological innovations, ecological needs, and circular economy objectives. The use of specialized microbial strains, capable of degrading organic pollutants or immobilizing heavy metals, demonstrates not only the potential to detoxify degraded soils, but also the ability to restore essential ecological functions. Examples documented in recent years, such as the application of bacterial consortia to decontaminate land affected by persistent pesticides, illustrate how these interventions can be scaled up without compromising local biodiversity [62,127,178,180,181].

In the field of bioremediation, microorganisms demonstrate high efficiency in degrading organic pollutants and mobilizing or immobilizing toxic elements, offering natural solutions for restoring affected agricultural ecosystems. In parallel, biofertilization through the use of nitrogen-fixing, phosphate-solubilizing, or phytohormone-producing bacteria has led to increased soil fertility and reduced dependence on chemical fertilizers. These processes not only improve agricultural productivity but also contribute to maintaining soil health in the long term, an essential aspect in the context of climate change and accelerated land degradation. Biofuel production utilizing microbial biomass, algae, and agricultural waste has evolved from simple fermentation processes to integrated platforms capable of generating bioethanol, biogas, biodiesel, or biohydrogen. These biotechnological routes offer viable alternatives to fossil fuels, reducing greenhouse gas emissions and diversifying the rural energy mix. At the same time, the development of microbial biochemical synthesis has expanded the range of products obtained from renewable sources, including biodegradable biopolymers, nutraceutical compounds, vitamins, functional ingredients, and microbial proteins, reinforcing the principles of the circular economy [21,34,35,163,182].

On the biofuel production front, microorganisms such as microalgae and cyanobacteria offer versatile biochemical routes for obtaining biodiesel, bioethanol, or biogas, utilizing unconventional resources, including wastewater and agricultural waste. Although current yields require optimizations at the metabolic and process engineering level, the integration of these technologies in mixed farms or agro-industrial parks can generate multiple benefits, from renewable energy to reducing waste management costs [84,120,183,184,185,186].

An emerging area with potential for disruption is next-generation food systems, which combine precision fermentation with metabolic engineering to produce microbial proteins, alternative fats, and customized ingredients. These systems offer clear advantages in terms of resource efficiency, emissions reduction, and production stability, but they also face significant challenges: technological barriers to scaling, high production costs, consumer reluctance, and the need for an adapted regulatory framework. Supportive policies, investments in biotechnology infrastructure, and public education campaigns are key factors that can accelerate their adoption [4,5,7,114,187,188].

In addition to the diversity of applications presented, the implementation of microorganisms in the transition to sustainable agriculture offers particular benefits. They can replace traditional chemical inputs, reducing soil and water pollution, while improving soil health and fertility in the long term. Through their ability to valorize agro-industrial waste and transform it into valuable products, these systems directly contribute to the circular economy and reduce the carbon footprint of agricultural supply chains. Moreover, innovations such as precision fermentation and microbial protein production diversify food sources that can increase the resilience of agri-food systems to climate change. However, large-scale implementation is limited by high infrastructure costs, the complexity of industrial scaling processes, and the lack of internationally harmonized legislative frameworks. In the field of biofertilizers, for example, practical issues such as the short shelf life of the products due to high temperatures during transportation and storage can reduce their effectiveness upon application [71]. Also, the formulation of microbial inoculants often faces constraints that affect their performance under real field conditions [189] xff. These challenges require further research and development to improve the stability and efficiency of biofertilizers in various agricultural environments. Public acceptance remains a critical factor, being influenced both by perceptions of the safety of the products obtained and by the lack of adequate scientific communication campaigns. These challenges highlight the need for an integrated approach, in which technological progress is supported by coherent public policies, industrial partnerships, and educational programs aimed at informing consumers [154,155,156].

To strengthen the coherence of the analysis, it is essential to highlight the interaction between biotechnology, ecology, and public policy, which form a synergistic framework for the implementation of microbial solutions. Biotechnology provides the tools to optimize microbial strains and production processes, ecology ensures the adaptation of these solutions to ecosystem dynamics and long-term sustainability, and public policy creates the legislative and economic framework necessary for large-scale implementation. For example, biofertilization strategies need to be calibrated in terms of both microbial performance and impact on soil biodiversity, and their adoption is accelerated by policies supporting circular agriculture. Similarly, the development of microbial biofuels cannot be separated from emission reduction objectives and market regulations for renewable energy. Integrating these fields not only ensures scientific coherence but also increases the chances of success in the transition to sustainable agri-food systems.

**Figure 11:**
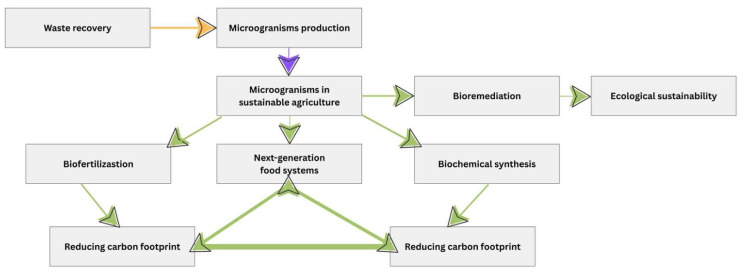
Role of microorganisms in sustainable agriculture.

In a general overview, microorganisms can no longer be perceived only as auxiliary agents in agriculture, but as pillars of a systemic transition to a sustainable bioeconomy (Figure 11). The link between their ecological functions, technological performance, and economic benefits outlines a paradigm in which agriculture, energy, the chemical industry, and the food sector are interconnected in a regenerative value chain. The future of these applications depends on an integrated approach, in which scientific research, technological innovation, and adaptive governance complement each other, paving the way towards resilient, efficient, and equitable production systems.

**Figure 12:**
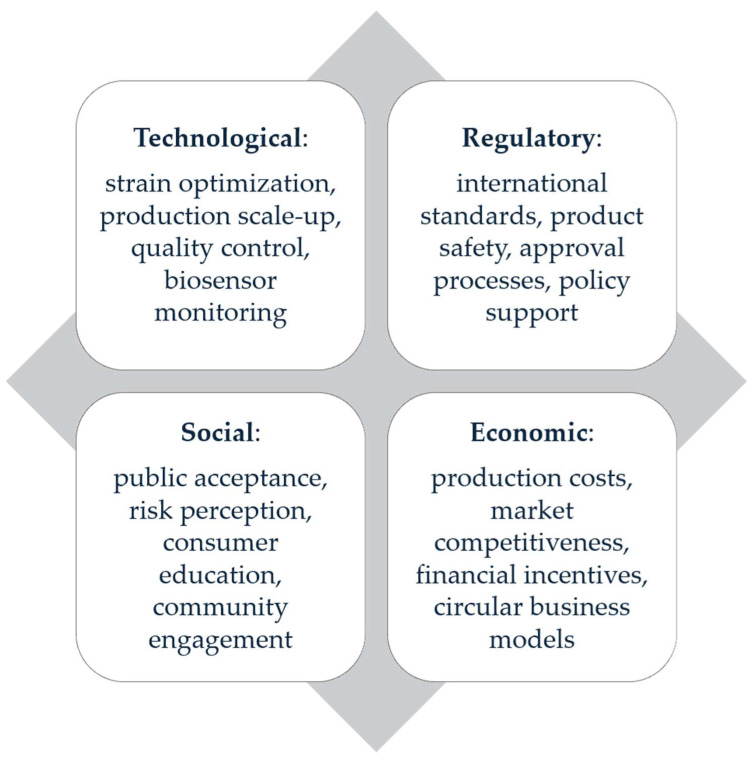
Dimensions of microorganism adoption in food systems.

The adoption of microorganisms in agri-food systems is influenced by a complex interaction between technological, regulatory, social, and economic dimensions, each of which plays a key role in determining the success of implementation. From a technological point of view, progress in the optimization of microbial strains, the development of scalable fermentation processes, and the use of biosensors for monitoring allow for increased efficiency and traceability of microbial interventions. However, these innovations must be accompanied by a coherent regulatory framework that ensures product safety, international standardization, and clear approval procedures, reducing uncertainty for economic actors. The social dimension is equally critical, as public acceptance and risk perception can accelerate or block large-scale adoption, which implies the need for education campaigns and the involvement of communities in decision-making processes. Added to these is the economic dimension, in which production costs, market competitiveness, and the existence of financial incentives can determine the commercial viability of microbial solutions. Integrating these four perspectives creates a holistic framework that not only facilitates the transition to sustainable food systems but also guides research and public policies towards common goals of resilience and efficiency. To provide an integrated and future-oriented perspective, Figure 12 illustrates the main technological, regulatory, social, and economic dimensions influencing the adoption of microorganisms in food systems, highlighting their interdependencies and underlining the need for interdisciplinary strategies. This visual synthesis not only supports the discussion of barriers and enablers but also indicates promising research directions for accelerating the transition to sustainable agri-food systems.

## 5. Conclusions

The literature review from 2005 to 2025 highlights substantial progress in the use of microorganisms as key tools for enhancing sustainability in agriculture, through their application in biofertilization, biological pest control, advanced biochemical synthesis, and the development of new generation food systems, such as precision fermentation and microbial proteins. The major advantages identified include reducing dependence on chemical inputs, converting agro-industrial waste into valuable products, increasing resource efficiency, diversifying food sources, and reducing environmental impact. In contrast, significant challenges relate to high production costs, technological barriers to scaling, limited consumer acceptance, and the lack of adapted legislative frameworks.

The novelty of this study lies in the integration of an interdisciplinary perspective, correlating biotechnological innovations with the economic, political, and social factors that condition their large-scale implementation. This approach allows us not only to understand their technological potential but also to identify critical points in the practical adoption of these solutions.

Through their ability to transform renewable resource flows into food products, biopolymers, or value-added compounds, microorganisms are emerging as strategic links in the transition towards a sustainable, resilient, and circular economy-oriented agriculture. Future research should aim at optimizing processes to reduce energy costs, increase yields, and develop flexible biotechnological platforms, capable of responding rapidly to climate change and global market demands.

## 6. Future Perspectives

The rapid development of microbial technologies over the past two decades highlights their transformative potential in agriculture, bioenergy, and innovative food systems, and clear strategic directions for research, policy, and industry are needed to fully harness these benefits. Future research should focus on optimizing microbial processes to reduce energy consumption, increase yields, and diversify substrates, and on developing modular and flexible biotechnological platforms capable of responding rapidly to climate change and global market demands, while integrating interdisciplinary perspectives that link microbiology with agronomy, economics, and social factors to better understand barriers to adoption. At the policy level, it is essential to establish clear and harmonized legislative frameworks for microbial products, provide incentives and funding for research and pilot implementation of sustainable technologies, and develop consumer education campaigns to increase public acceptance of products derived from microorganisms. The industry should adopt effective strategies for scaling up production, integrate circular economy principles through waste valorization, and support collaborative opportunities between academia, industry, and governments to accelerate the adoption of microbial innovations, promoting technology transfer and rapid market entry. By coordinating efforts across research, policy, and industry and leveraging strategic collaborations, microbial technologies can lead to sustainable, resilient, and innovation-driven agriculture, maximizing technological potential and addressing economic, social, and environmental challenges.

## Figures and Tables

**Table 1:** Role of microorganisms in the bioremediation process.

Microorganism	Role in Bioremediation	Example	Key Takeaways	Refs.
hydrocarbon degrading bacteria	metabolize petroleum hydrocarbons and transform them into less toxic compounds	*Pseudomonas* *Alcanivorax*	efficiently degrades petroleum hydrocarbons, reduces environmental toxicity, contributes to soil regeneration	[50,52]
metal-reducing bacteria	reduce and immobilize heavy metals in contaminated soils and waters	*Geobacter* *Shewanella*	facilitate heavy metal detoxification	[53,54]
fungi and yeasts	degrade complex organic compounds and pollutants	*Aspergillus* *Candida*	improves soil and wastewater bioremediation	[55,56]
cyanobacteria	absorb and fix inorganic pollutants, contributing to wastewater treatment	*Anabaena* *Nostoc*	removes nutrients and pollutants from wastewater, contributes to nitrogen fixation	[57]
microbial consortia	synergistic action of multiple species for simultaneous degradation of mixed pollutants	mixed bacteria–fungi consortia in contaminated soils	pollutant degradation due to synergistic metabolic interactions among species	[58,59,60]

**Table 2:** Role of microorganisms in the biofertilization process.

Microorganism	Role in Bioremediation	Example	Key Takeaways	Refs.
nitrogen-fixing bacteria	convert atmospheric nitrogen (N_2_) into ammonia, making it available to plants and improving soil fertility	*Rhizobium* *Azotobacter* *Azospirillum*	enhance soil nitrogen content, support plant growth, reduce the need for chemical fertilizers	[74,75,76]
phosphate-solubilizing bacteria	release insoluble phosphates in soil through organic acid production, enhancing plant phosphorus uptake	*Pseudomonas* *Bacillus*	improves phosphorus bioavailability, promotes better plant nutrition and increased crop yields	[77]
mycorrhizal fungi	form symbiotic associations with plant roots, improving water and nutrient uptake, especially phosphorus	*Glomus* *Gigaspora*	enhance plant nutrient, water uptake, improve plant growth and stress resilience	[44,74]
potassium-solubilizing microorganisms	mobilize potassium from insoluble minerals, increasing plant growth and stress tolerance	*Bacillus mucilaginosus* *Aspergillus niger*	increase potassium availability, improve plant development and tolerance to abiotic stress	[75,76,78]
plant growth-promoting rhizobacteria	produce phytohormones (auxins, gibberellins, cytokinins), suppress pathogens, and enhance root growth	*Burkholderia* *Enterobacter*	promote root development, enhance plant resistance to pathogens, stimulate plant growth	[79]

**Table 3:** Role of microorganisms in the biofuel production process.

Biofuel	Microorganisms	Role	Examples	Key Takeaways	Refs.
bioethanol	bacteria and yeasts	fermentation of sugars to produce ethanol	*Saccharomyces cerevisiae* *Zymomonas mobilis*	efficiently converts sugars into ethanol, provides a scalable and renewable biofuel source	[102]
biodiesel	algae and cyanobacteria	accumulation of lipids that can be converted into biodiesel	*Chlorella vulgaris* *Nannochloropsis* *Spirulina platensis*	high lipid content, sustainable alternative to fossil diesel	[103,104,105]
biogas	methanogenic and anaerobic bacteria	anaerobic decomposition of organic matter to produce CH_4_ (methane)	*Methanobacterium* *Methanosarcina* anaerobic microbial consortia	converts organic waste into methane, supports waste-to-energy strategies	[101,102]
biohydrogen	bacteria and photosynthetic algae	photobiological or fermentative production of H_2_ (hydrogen) from organic substrates or light	*Clostridium* *Rhodobacter sphaeroides* *Chlamydomonas reinhardtii*	produces hydrogen sustainably, provides a clean energy alternative	[106,107,108]
biobutanol	acetobutylic bacteria	fermentation of carbohydrates to produce butanol	*Clostridium acetobutylicum Clostridium beijerinckii*	produces butanol efficiently, offers higher energy density than ethanol	[82,109]
microbial biomass (feedstock)	bacteria and yeasts	produced biomass can be further converted into solid or liquid fuels	*Escherichia coli Saccharomyces cerevisiae*	serves as a versatile feedstock for biofuels, enables multiple conversion pathways and added value	[106,110,111,112]

**Table 4:** Microorganisms in biochemical synthesis for sustainable agriculture.

Product	Role/Application	Microorganisms	Key Takeaways	Refs.
biodegradable polymers	alternative to petroleum-based plastics, plastic waste reduction	*Cupriavidus necator* *Halomonas*	sustainable alternative to plastics, reduce environmental pollution, and support circular economy approaches	[139]
nutraceuticals and pharmacological compounds	production of resveratrol, naringenin, and curcuminoids, use of agro-industrial waste	*Saccharomyces cerevisiae* *E. coli*	high-value nutraceuticals from renewable substrates, provide scalable and sustainable production routes	[133,134]
vitamin precursors	intermediate for vitamin C production, scalable, efficient bioprocesses	*Gluconobacter oxydans* *Ketogulonicigenium* *B. megaterium*	enable cost-effective and scalable production of vitamin precursors, enhance industrial supply, reduce chemical synthesis dependency	[140]
waste-to-protein systems	conversion of agro-industrial residues into microbial protein for animal feed	mixed microbial consortia	convert agro-industrial waste into protein-rich biomass, support sustainable animal nutrition and waste valorization	[117,141,142,143]
fermentative biopolymers	production of exopolysaccharides (xanthan, pullulan, curdlan, bacterial cellulose) for food and industrial applications	*Aspergillus* *Bacillus* *Xanthomonas* *Aureobasidium*	provide functional biopolymers for food and industrial use, offer environmentally friendly alternatives to synthetic polymers	[136,137]

**Table 5:** Main advantages and disadvantages of microorganisms in sustainable agriculture.

Application Area	Advantages	Disadvantages
bioremediation	degradation of toxic pollutants into less harmful compounds, eco-friendly alternative to chemical methods, cost-effective for large-scale contaminated sites	slow degradation rates for some pollutants, sensitivity to environmental conditions, incomplete mineralization can generate secondary products
biofertilization	improved nutrient availability (N, P, K), enhanced soil health and fertility, reduced dependence on chemical fertilizers, and promotion of plant growth	variable efficiency under field conditions, competition with native soil microbiota, limited shelf life of microbial inoculants
biofuel production	renewable energy source, potential use of agro-industrial waste as feedstock, reduction in greenhouse gas emissions	high production costs, difficulties in large-scale scalability, technical barriers in downstream processing
biochemical synthesis	production of biodegradable polymers, nutraceuticals, vitamins, and bioplastics, valorization of agricultural residues, contribution to circular economy	low yields for some target compounds, complex metabolic engineering required, regulatory and safety concerns for new bioproducts
next-generation food systems (precision fermentation, microbial protein)	sustainable protein alternatives, reduced land and water use, decoupling from traditional livestock, alignment with climate goals	consumer acceptance issues, high production costs, strict regulatory frameworks, scale-up and infrastructure limitations

**Table 6:** Comparative table regarding microbial products that are either commercialized or in pilot phases.

Category	Commercialized Product	Pilot-Stage Product	Refs.
Biofuel	ethanol from fermentation	hydrogen from microbial fermentation (e.g., *Clostridium* spp.)	[171,172]
Microbial protein	microalgae proteins (e.g., *Spirulina*, *Chlorella*)	single-cell proteins from methanotrophic bacteria	[171,172,173]
Pigment	carotenoids (e.g., β-carotene, astaxanthin) from fungal and bacterial cultures	pigments from metal-tolerant bacterial cultures	[174,175]
Bioplastic	polyhydroxyalkanoates (PHAs) from bacterial cultures	PHAs from methanotrophic bacteria or organic waste	[136]
Biofertilizer	*Azotobacter*, *Rhizobium*, and *Bacillus* spp. for agriculture	microorganisms for bioremediation of soils contaminated with heavy metals or pesticides	[67,79,176]
Food ingredient	probiotic bacteria (*Lactobacillus*, *Bifidobacterium*)	microbial proteins from methanotrophic bacteria for alternative foods	[172,177]

## Data Availability

The original contributions presented in this study are included in the article. Further inquiries can be directed to the corresponding author.
